# EZH2 Mediates miR-146a-5p/HIF-1*α* to Alleviate Inflammation and Glycolysis after Acute Spinal Cord Injury

**DOI:** 10.1155/2021/5591582

**Published:** 2021-05-19

**Authors:** Shuangfei Ni, Bo Yang, Lei Xia, Huafeng Zhang

**Affiliations:** ^1^Orthopaedics, The First Affiliated Hospital of Zhengzhou University, Zhengzhou City, Henan Province, China; ^2^Neurosurgery, The First Affiliated Hospital of Zhengzhou University, China

## Abstract

Acute spinal cord injury (ASCI) is a severe traumatic disease of the central nervous system, the underlying mechanism of which is unclear. This study was intended to study the role of EZH2 and miR-146a-5p/HIF-1*α* in inflammation and glycolysis after ASCI, providing reference and basis for the clinical treatment and prognosis of ASCI injury. We used lipopolysaccharide (LPS) to induce inflammation of microglia, and we constructed the ASCI animal model. qRT-PCR detected the relative expression levels of EZH2, HIF-1*α*, miR-146a-5p, IL-6, TNF-*α*, IL-17, PKM2, GLUT1, and HK2 in cells and tissues. Western blot was performed to detect the expression levels of EZH2, HIF-1*α*, H3K27me3, IL-6, TNF-*α*, IL-17, PKM2, GLUT1, and HK2. ChIP verified the enrichment of H3K27me3 in the miR-146a-5p promoter region. Bioinformatics predicted the binding sites of HIF-1*α* and miR-146a-5p, and dual-luciferase reporter assay verified the binding of HIF-1*α* and miR-146a-5p. ELISA detects the levels of inflammatory factors IL-6, TNF-*α*, and IL-17 in the cerebrospinal fluid of rats. The GC-TOFMS was used to detect the changes of glycolytic metabolites in the cerebrospinal fluid of rats. EZH2 could mediate inflammation and glycolysis of microglia. EZH2 regulates inflammation and glycolysis through HIF-1*α*. EZH2 indirectly regulated the HIF-1*α* expression by mediating miR-146a-5p. EZH2 mediates miR-146a-5p/HIF-1*α* to alleviate inflammation and glycolysis in ASCI rats. In the present study, our results demonstrated that EZH2 could mediate miR-146a-5p/HIF-1*α* to alleviate the inflammation and glycolysis after ASCI. Therefore, EZH2/miR-146a-5p/HIF-1*α* might be a novel potential target for treating ASCI.

## 1. Introduction

Acute spinal cord injury (ASCI) is a severe traumatic disease of the central nervous system (CNS) [1]. Spinal cord tissues respond to injury after ASCI, and the gene expression profile of cells changes, which affects the repair of injury after ASCI, and epigenetic regulation plays an important role. Enhancer of zeste homology 2 (EZH2) is located on the human chromosome 7q35. EZH2 mainly catalyzes the histone H3 lysine 27 trimethylation (H3K27me3), which mediates the change of chromatin structure, closes transcription factor binding sites, inhibits the transcription of target genes, and participates in many physiological or pathological processes [2]. Studies have shown that nerve damage dramatically increases the number of microglia expressing EZH2 [3]. Therefore, it is an effective strategy to improve the functional recovery after ASCI by exploring the means of regulating the orderly response of cell genes, thus promoting angiogenesis.

Hypoxia inducible factor 1 (HIF-1) is a heterodimer, which is composed of HIF-1*α* and HIF-1*β*. The expression of HIF-1*α* is regulated by cell oxygen concentration and plays a very important role in immune response [4]. HIF-1*α* expression increases early after ASCI and plays a protective role in nerve recovery [5]. Hypoxia could enhance HIF-1*α* expression in microglia and promote microglia migration [6]. Studies have found that under high glucose-stimulated human peritoneal mesothelial cells, EZH2 could increase the trimethylation of H3K4 on the HIF-1*α* promoter and directly activate the transcription of HIF-1*α* [7]. It is reported that HIF-1 is the main regulator of glycolysis [8]. Spinal cord injury (SCI) can lead to rapid muscle atrophy and oxidative-glycolytic fiber-type transformation. Acute disruption of skeletal muscle glucose uptake 7 days after ASCI leads to decreased pyruvate and lactate levels [9]. However, there are few studies on the inflammation and glycolysis mediated by EZH2 and HIF-1*α* after ASCI.

MicroRNA (miRNA), as a highly conserved short noncoding RNA, plays an important role in a lot of biological functions (tissue development, organogenesis, and metabolism) [10]. Many studies have reported that a large number of miRNAs are dysregulated after ASCI, indicating that miRNAs might be involved in the regulation of ASCI [11, 12]. As an important type of miRNA, miR-146a participates in regulating immune and inflammation processes [13]. It has been shown that miR-146a-5p can alleviate neuropathic pain in the spinal cord [14, 15]. Studies also reported that miR-146a-5p is downregulated after ASCI [16], and studies have also verified that miR-125b-5p/miR-146a-5p targeting the expression of HIF-1/SP1-dependent ROBO4 may delay the progression of diabetic retinopathy [17]. However, little research has been done on the regulatory relationship between HIF-1*α* and miR-146a-5p in ASCI.

Therefore, the present study focused on the regulatory mechanisms of EZH2 and miR-146a-5p/HIF-1*α* in inflammation and glycolysis after ASCI. Through the above studies, we intend to elucidate the possibility of EZH2-miR-146a-5p/HIF-1*α* as a new target for treating ASCI and other inflammation-related CNS injuries, so as to provide reference and basis for the clinical treatment and prognosis of ASCI injury.

## 2. Material and Methods

### 2.1. Cell Culture and Transfection

Microglia were purchased from HonorGene (Changsha, China). Microglia were cultured in DMEM (#41500034, GIBCO, China) medium containing 10% FBS (#10099141, GIBCO, China), placed at 37°C, and cultivated in a humidity chamber with 5% CO_2_. Lipopolysaccharide (LPS) induces inflammation of microglia [18], which is divided into two groups: Control group and LPS group (given with a final concentration of 1.0 *μ*g/mL LPS) [19, 20]. The short hairpin targeting EZH2 (sh-EZH2) and sh-NC were synthesized by Sangon Biotech (Shanghai, China) for knockdown EZH2 expression. To overexpress HIF-1*α*, HIF-1*α* sequences were ligated with the LV003 vector.

### 2.2. Establishment of the ASCI Model

Forty-eight SD rats (200-250 g) were randomly divided into a Sham group, ASCI group, ASCI+NC-EZH2 group, and ASCI+sh-EZH2 group. The latter three groups were collectively referred to as the model group, with 12 rats in each group. The method of constructing the ASCI animal model was as follows: after conventional anesthesia, rats were placed in the prone position and skin preparation, disinfection, and sheet laying were carried out. Then, a longitudinal incision was made with the spinous process of thoracic 10 as the center. The skin, subcutaneous, and muscle were cut in turn, and the vertebral plate was removed to expose the spinal cord. The modified Allen's strike method [21] was used to establish an incomplete spinal cord contusion model (the strike force was 8 g × 25 mm). Hemorrhage and swelling of the spinal cord tissue and spastic twitching of the lower limbs of the rats indicate that the animal model was successfully constructed. After washing the wound, we closed the incision layer by layer. Penicillin 2WU/Bid was routinely injected intramuscularly to prevent infection. After the operation, we massaged the bladder regularly to help urinate, 2-4 times a day, until the rats could urinate spontaneously. In the Sham group, the wound was closed immediately after the vertebral plate resection, and the spinal cord was not exposed to any mechanical injury. 5 min after the rat model was established, sh-EZH2 was transfected into the rat. After 72 h of the animal model, the rats were sacrificed and the blood and cerebrospinal fluid collected.

### 2.3. Basso-Beattie-Bresnahan (BBB) Score Assessed Hindlimb Function in Rats

The rats were all subjected to behavioral tests for the recovery of hindlimb function at 12, 24, and 48 h before injury and after treatment, with BBB score as an indicator [22]. We observed the motion of the ankle joint of the hindlimbs, landing of the sole and instep of the foot, stability of the trunk, and position of the tail.

### 2.4. Quantitative Real-Time PCR (qRT-PCR).

qRT-PCR was used to test the relative expression levels of EZH2, HIF-1*α*, miR-146a-5p, IL-6, TNF-*α*, IL-17, PKM2, GLUT1, and HK2 in cells and tissues. In short, total RNA was extracted by TRIzol method, and RNA was reversely transcribed into cDNAs in accordance with the instruction of a reverse transcription kit (CW2569, CWBIO, China). The relative expression of genes was tested by SYBR Green qPCR mix (Invitrogen) on an ABI 7900 system. The internal reference gene was *β*-actin, and the 2^-*ΔΔ*Ct^ method was used to calculate the relative transcription level of the target gene. The primer sequences used in this study are shown in Table 1.

### 2.5. Western Blot

The protein was extracted from tissues and cells with a RIPA lysis buffer (#P0013B, Beyotime Biotechnology) and mixed with a SDS-PAGE loading buffer (#MB2479, Meilunbio). The mixture was heated in boiling water at 100°C for 5 min. The membrane was blocked with 5% skim milk for 2 h at room temperature and incubated with the primary antibody EZH2 (#21800-1-AP, dilution 1 : 1500, Proteintech), HIF-1*α* (#ab1, dilution 1 : 400, Abcam), H3K27me3 (#ab9050, dilution 1 : 1000, Abcam), IL-6 (#bs-0782R, dilution 1 : 1000, Bioworld), TNF-*α* (#ab6671, dilution 1 : 1000, Abcam), IL-17 (#ab79056, dilution 1 : 1000, Abcam), PKM2 (#15822-1-AP, dilution 1 : 2000, Proteintech), GLUT1 (#21829-1-AP, dilution 1 : 1000, Proteintech), HK2 (#ab209847, dilution 1 : 1500, Abcam), and *β*-actin (#66009-1-Ig, dilution 1 : 2000, Proteintech) at 4°C. Rinse three times with TBST and incubate with secondary antibodies. The protein bands were detected by the ChemiScope 6100 system (Clinx Co., Ltd., Shanghai, China); the internal reference for expression levels was *β*-actin.

### 2.6. Chromatin Immunoprecipitation (ChIP)

We used a ChIP kit to verify the enrichment of H3K27me3 in the miR-146a-5p promoter region. Cells were fixed with 1% formalin for 10 minutes, and the DNA was randomly fragmented to 200-800 bp by ultrasound, and the DNA was immunoprecipitated with a specific antibody against the target protein of H3K27me3 (#ab32106, dilution 1 : 1000, Abcam). Then, we use 100 *μ*L H_2_O for purification and elution of ChIP DNA, and qRT-PCR detected 2.5 *μ*L ChIP-DNA [7]. Different primers were used to detect the enrichment of H3K27me3 in the miR-146a-5p promoter region. Primer sequences used are shown in Table 1.

### 2.7. Bioinformatics Prediction and Dual-Luciferase Assay

We used starBase to predict the binding site of HIF-1*α* and miR-146a-5p. To verify the binding of HIF-1*α* to miR-146a-5p, wild-type (WT) or mutant (MUT) HIF-1*α* fragments were constructed and inserted into a pmirGLO Vector (Promega). We use the Lipofectamine 3000 reagent (Thermo Fisher Scientific) with transfected recombinant vectors into MG. NC and miR-145-5p mimics were simultaneously introduced into the cells as specified. Then, we measured the luciferase activity [23].

### 2.8. ELISA

The cerebrospinal fluid of rats was tested for inflammatory factors. The inflammatory factors IL-6, TNF-*α*, and IL-17 were detected with a quantitative IL-6, TNF-*α*, and IL-17 ELISA kit according to the manufacturer's instructions. The concentration of IL-6, TNF-*α*, and IL-17 in cerebrospinal fluid was calculated by the American DYNATECH MR 7000 microplate reader to form a standard curve with the provided values, and the results were expressed in pg/mL.

### 2.9. Metabolomics Analysis

Gas Chromatography Time-of-Flight Mass Spectrometry (GC-TOFMS) was used to detect glycolytic metabolite Glucose-6-Phosphate (G6P), Fructose-6-Phosphate (F6P), SUCCINATE, and MALATE changes in the cerebrospinal fluid of rats, and they were divided into four groups: sham, ASCI, ASCI+Nacl, and ASCI+GSK126 (EZH2 inhibitor).

### 2.10. Statistical Analysis

The statistical analysis of the data was performed using GraphPad 8.0. Three independent experimental data were expressed as mean ± standard deviation (SD). Differences between two or more groups were analyzed using Student's *t*-test or using one-way ANOVA. *P* < 0.05 was considered statistically significant.

## 3. Results

### 3.1. The Inflammation, Glycolysis, and HIF-1*α* in Microglia Treated with LPS Were Associated with Upregulated EZH2

**Figure 1:**
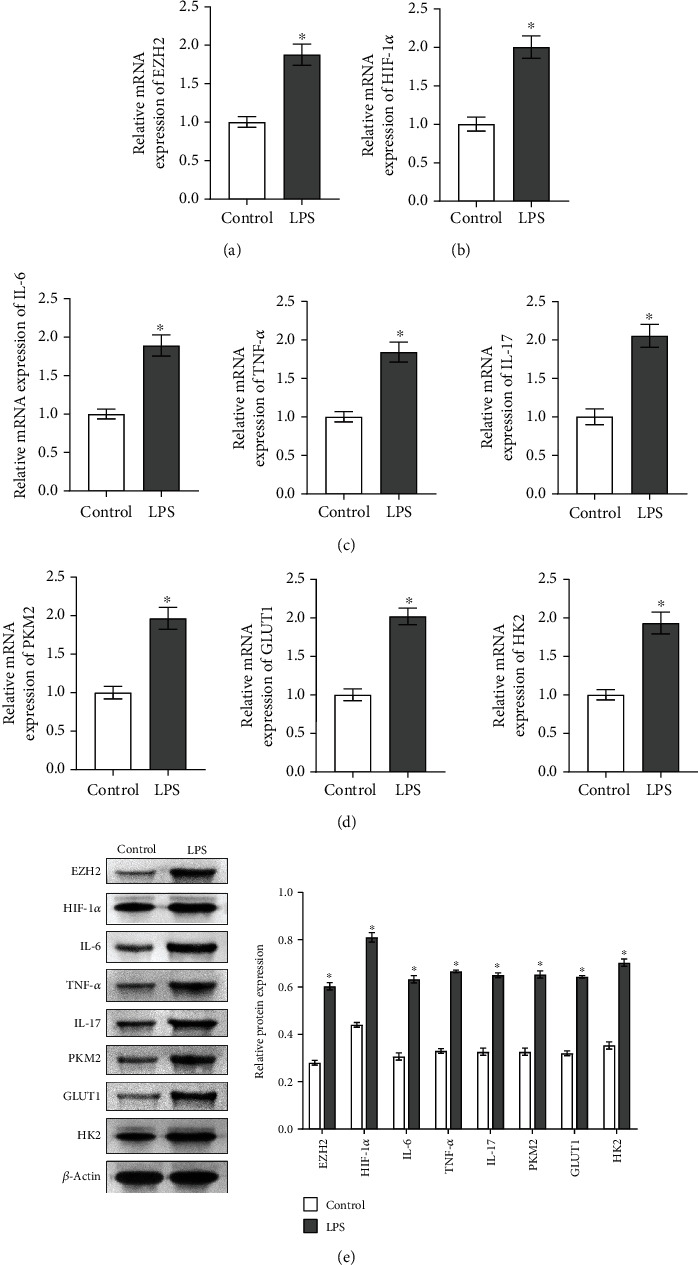
The inflammation, glycolysis, and HIF-1*α* in microglia treated with LPS were associated with upregulated EZH2. In the control group and LPS group. (a) qRT-PCR detected EZH2 expression. (b) qRT-PCR was performed to detect the expression of HIF-1*α*. (c) The expressions of inflammatory factors IL-6, TNF-*α*, and IL-17 were detected by qRT-PCR. (d) qRT-PCR detected glycolysis-related gene PKM2, GLUT1,and HK2 expressions. (e) Western blot analysis of the expression of EZH2; HIF-1*α*; inflammatory factors IL-6, TNF-*α*, and IL-17; and glycolysis-related genes PKM2, GLUT1, and HK2. ^∗^*P* < 0.05 vs. Control group.

In order to study the inflammation, glycolysis, and relationship between HIF-1*α* and EZH2 in microglia treated with LPS, qRT-PCR and Western blot were performed to detect EZH2; HIF-1*α*; inflammatory factors IL-6, TNF-*α*, and IL-17; and glycolysis-related gene PKM2, GLUT1, and HK2 expression levels in the Control and LPS groups. qRT-PCR and Western blot showed that compared with the Control group, EZH2 and HIF-1*α* expression in the LPS group was increased (fold change ≈ 2; ^∗^*P* < 0.05 vs. Control group; Figures 1(a), 1(b), and 1(e)). The expression of inflammatory factors IL-6, TNF-*α*, and IL-17 and glycolysis-related genes PKM2, GLUT1, and HK2 also increased in the LPS group (fold change ≈ 1.5 ~ 3; ^∗^*P* < 0.05 vs. Control group; Figures 1(c)–1(e)), which indicated that HIF-1*α* inflammation and glycolysis in microglia treated with LPS were associated with upregulated EZH2.

### 3.2. The Inflammation and Glycolysis in Microglia Were Mediated by EZH2

**Figure 2:**
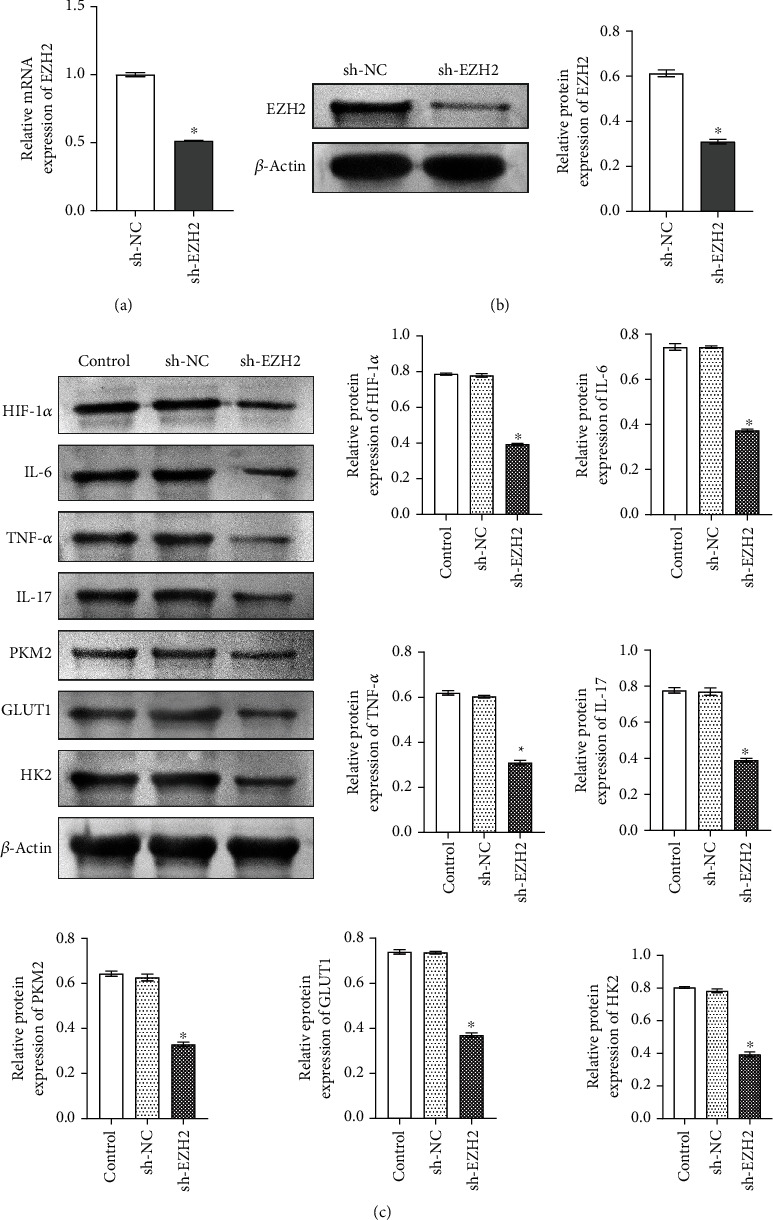
The inflammation and glycolysis in microglia were mediated by EZH2. (a, b) qRT-PCR and Western blot detected the knockdown efficiency of EZH2 in MG, respectively. ^∗^*P* < 0.05 vs. sh-NC group. (c) Western blot was performed to detect HIF-1*α*; inflammatory factors IL-6, TNF-*α*, and IL-17; and glycolysis-related gene PKM2, GLUT1, and HK2 expressions in the Control group, sh-NC group, and sh-EZH2 group. ∗*P* < 0.05 vs. Control group.

To investigate whether EZH2 is related to the inflammation and glycolysis of microglia, we knocked down EZH2 in microglia. As shown in Figures 2(a) and 2(b), qRT-PCR and Western blot results show the knockdown efficiency of EZH2 in microglia (fold change ≈ 0.5; ^∗^*P* < 0.05 vs. sh-NC group). Compared with the Control group and the sh-NC group, HIF-1*α*; inflammatory factors IL-6, TNF-*α*, and IL-17; and glycolysis-related gene PKM2, GLUT1, and HK2 expressions decreased in the sh-EZH2 group (fold change ≈ 0.5; ^∗^*P* < 0.05 vs. Control group; Figure 2(c)), indicating that EZH2 could mediate inflammation and glycolysis of microglia.

### 3.3. EZH2 Regulated Inflammation and Glycolysis through HIF-1*α*

**Figure 3:**
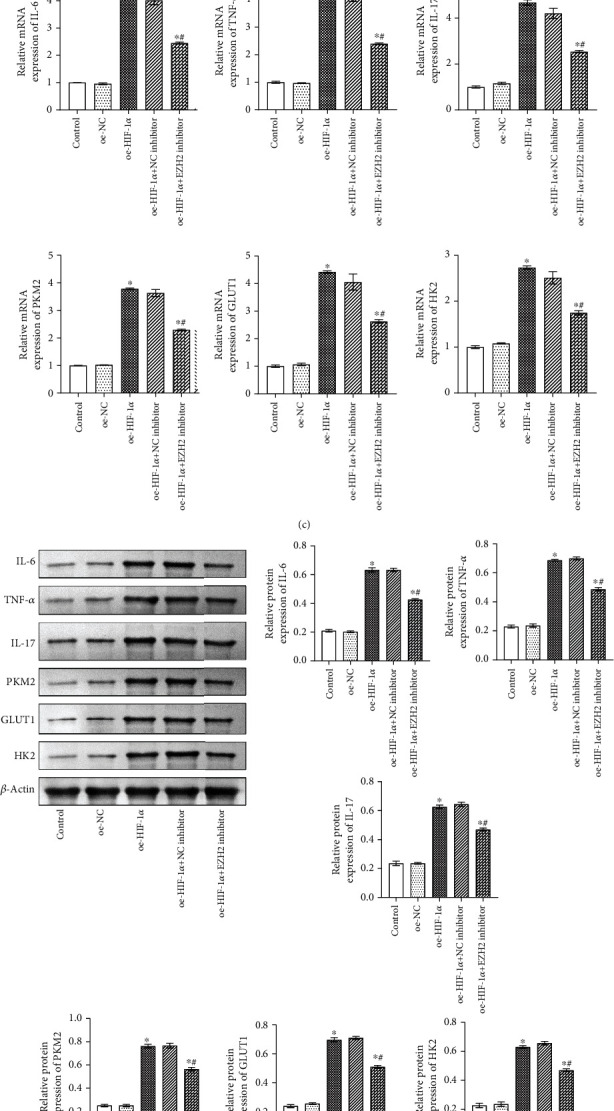
EZH2 regulated inflammation and glycolysis through HIF-1*α*. (a, b) qRT-PCR and Western blot were performed to detect the overexpression of HIF-1*α* in microglia, respectively. ^∗^*P* < 0.05 vs oe-NC group. (c) qRT-PCR were used to detect inflammatory factors IL-6, TNF-*α*, and IL-17 and glycolysis-related gene PKM2, GLUT1, and HK2 expressions. ^∗^*P* < 0.05 vs Control group; ^#^*P* < 0.05 vs oe-HIF-1*α* group. (d) Western blot detected inflammatory factors IL-6, TNF-*α*, and IL-17 and glycolysis-related gene PKM2, GLUT1, and HK2 expressions. ^∗^*P* < 0.05 vs. Control group; ^#^*P* < 0.05 vs. oe-HIF-1*α* group.

To study the relationship between EZH2 and HIF-1*α* in inflammation and glycolysis, we performed interference with EZH2 and overexpression of HIF-1 in microglia. As shown in Figures 3(a) and 3(b), the results of qRT-PCR and Western blot showed the overexpression efficiency of HIF-1*α* in microglia (fold change ≈ 2; ^∗^*P* < 0.05 vs. oe-NC group). The qRT-PCR and Western blot results showed that compared with the Control group and the oe-NC group, the expression levels of inflammatory factors IL-6, TNF-*α*, and IL-17 and glycolysis-related genes PKM2, GLUT1, and HK2 were increased in the oe-HIF-1*α* group, while decreased in the oe-HIF-1*α*+EZH2 inhibitor group (fold change ≈ 1.5 ~ 4.5; ^∗^*P* < 0.05 vs. Control group; ^#^*P* < 0.05 vs. oe-HIF-1*α* group; Figures 3(c) and 3(d)). These results indicate that EZH2 regulated inflammation and glycolysis through HIF-1*α*.

### 3.4. EZH2 Indirectly Regulated the HIF-1*α* Expression by Mediating miR-146a-5p

**Figure 4:**
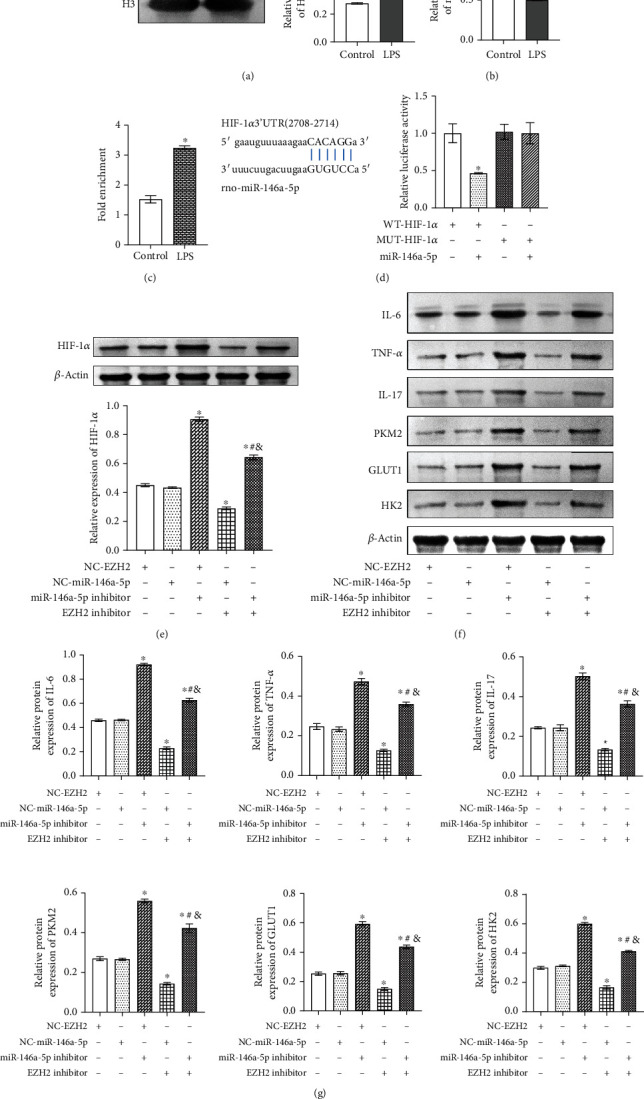
EZH2 indirectly regulated the HIF-1*α* expression by mediating miR-146a-5p. (a) Western blot detected the expression of H3K27me3 in the Control group and LPS group. ^∗^*P* < 0.05 vs. Control group. (b) qRT-PCR detected miR-146a-5p expression in the Control group and LPS group. ^∗^*P* < 0.05 vs. Control group. (c) ChIP was used to analyze the enrichment of H3K27me3 in the miR-146a-5p promoter region. ^∗^*P* < 0.05 vs. Control group. (d) starBase predicted the binding sites of HIF-1*α* and miR-146a-5p, and dual-luciferase report assay verified the binding of HIF-1*α* and miR-146a-5p. ^∗^*P* < 0.05 vs. WT-HIF-1*α* group. (e) Western blot detected the expression of HIF-1*α* in the NC-EZH2 group, NC-miR-146a-5p group, miR-146a-5p inhibitor group, EZH2 inhibitor+NC-miR-146a-5p group, and EZH2 inhibitor+miR-146a-5p inhibitor group. ^∗^*P* < 0.05 vs. NC-EZH2 group; ^#^*P* < 0.05 vs. NC-EZH2+miR-146a-5p inhibitor group; ^&^*P* < 0.05 vs. NC-miR-146a-5p+EZH2 inhibitor. (f) Western blot detected inflammatory factors IL-6, TNF-*α*, and IL-17 and glycolysis-related gene PKM2, GLUT1, and HK2 expressions. (g) Inflammatory factors IL-6, TNF-*α*, and IL-17 and glycolysis-related gene PKM2, GLUT1, and HK2 expressions. ^∗^*P* < 0.05 vs. NC-EZH2 group; ^#^*P* < 0.05 vs. NC-EZH2+miR-146a-5p inhibitor group; ^&^*P* < 0.05 vs. NC-miR-146a-5p+EZH2 inhibitor.

To explore the relationship between H3K27me3 and miR-146a-5p, we first performed Western blot and qRT-PCR verification in microglia treated by LPS. The Western blot results showed that H3K27me3 was upregulated in the LPS group compared with the Control group (fold change ≈ 3; ^∗^*P* < 0.05 vs Control group; Figure 4(a)). qRT-PCR results suggested that miR-146a-5p was downregulated in the LPS group compared with the Control group (fold change ≈ 0.5; ^∗^*P* < 0.05 vs Control group; Figure 4(b)). Subsequently, ChIP verified the enrichment of H3K27me3 in the miR-146a-5p promoter region, and the results showed that H3K27me3 occurred in miR-146a-5p (fold change ≈ 2; ^∗^*P* < 0.05 vs Control group; Figure 4(c)). To investigate the targeting relationship between miR-146a-5p and HIF-1*α*, starBase confirmed the binding sites of miR-146a-5p and HIF-1*α*. Dual-luciferase report assay showed that the luciferase activity of WT HIF-1*α* was significantly decreased after transfection of miR-145-5p mimics (fold change ≈ 0.5; ^∗^*P* < 0.05 vs WT-HIF-1*α* group; Figure 4(d)). These results suggested that miR-146a-5p binds to HIF-1*α*.

Furthermore, we used EZH2 and miR-146a-5p inhibitors to study whether EZH2 could regulate HIF-1*α* expression through miR-146a-5p. As shown in Figures 4(e)–4(g), compared with the NC-EZH2 group and the NC-miR-146a-5p group, HIF-1; inflammatory factors IL-6, TNF-*α*, and IL-17; and glycolysis-related gene PKM2, GLUT1, and HK2 expressions were upregulated in the miR-146a-5p inhibitor group and EZH2 inhibitor+miR-146a-5p inhibitor group, but downregulated in the EZH2 inhibitor+NC-miR-146a-5p group (fold change ≈ 0.5 ~ 2.5; ^∗^*P* < 0.05 vs. NC-EZH2 group; ^#^*P* < 0.05 vs. NC-EZH2+miR-146a-5p inhibitor group; ^&^*P* < 0.05 vs. NC-miR-146a-5p+EZH2 inhibitor), which confirmed that EZH2 indirectly regulated the HIF-1*α* expression by mediating miR-146a-5p.

### 3.5. EZH2 Mediates miR-146a-5p/HIF-1*α* to Alleviate Inflammation and Glycolysis in ASCI Rats

**Figure 5:**
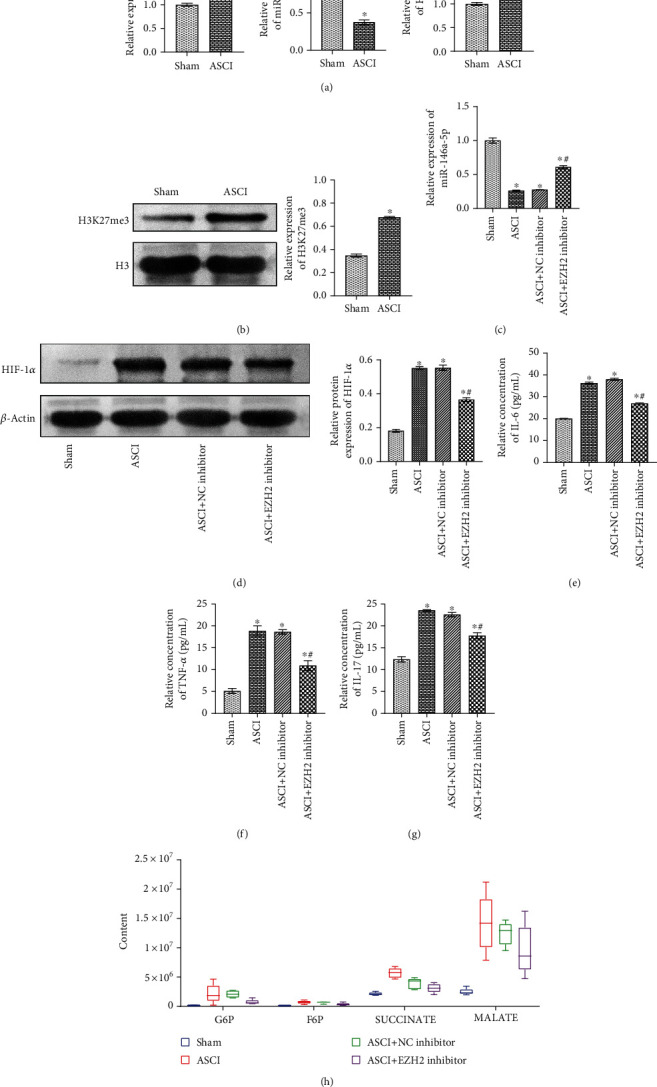
EZH2 mediates miR-146a-5p/HIF-1*α* to alleviate inflammation and glycolysis in ASCI rats. (a) qRT-PCR detected EZH2, miR-146a-5p, and HIF-1*α* expressions in the Sham group and ASCI group. (b) Western blot detected the expression of H3K27me3 in the Sham group and ASCI group. (c) qRT-PCR was performed to detect miR-146a-5p expression. (d) Western blot detected the expression of HIF-1*α*. (e–g) ELISA was performed to detect inflammatory factor IL-6, TNF-*α*, and IL-17 levels in the cerebrospinal fluid of rats. (h) Metabolomics detected the relative levels of glycolytic metabolites G6P, F6P, SUCCINATE, and MALATE. ^∗^*P* < 0.05 vs. Sham group; ^#^*P* < 0.05 vs. ASCI group.

We have demonstrated that EZH2 indirectly regulated the HIF-1*α* expression by mediating miR-146a-5p at the cellular level. To study the relationship between EZH2 and miR-146a-5p/HIF-1*α* in vivo, we constructed the ASCI animal model. The qRT-PCR results indicated that, compared with the Sham group, EZH2 and HIF-1*α* expression levels in the ASCI group were increased, while the expression levels of miR-146a-5p were decreased (fold change ≈ 0.3 ~ 2.5; ^∗^*P* < 0.05 vs Sham group; Figure 5(a)). Western blot results showed that compared with the Sham group, H3K27me3 expression was upregulated in the ASCI group (fold change ≈ 2; ^∗^*P* < 0.05 vs. Sham group; Figure 5(b)).

Subsequently, we investigated the regulatory effects of EZH2 and miR-146a-5p/HIF-1*α* on inflammation and glycolysis in the Sham group, ASCI group, ASCI+NC inhibitor group, and ASCI+EZH2 inhibitor group. The qRT-PCR results showed that miR-146a-5p expression in the ASCI group was decreased compared with that in the Sham group. Compared with the ASCI group and the ASCI+NC inhibitor group, miR-146a-5p expression in the ASCI+EZH2 inhibitor group was increased, which indicated that downregulation of EZH2 promoted the expression of miR-146a-5p in ASCI rats (fold change ≈ 0.3 ~ 0.7; ^∗^*P* < 0.05 vs. Sham group; ^#^*P* < 0.05 vs. ASCI group; Figure 5(c)). The Western blot results showed that compared with the Sham group, HIF-1*α* expression was increased in the ASCI group. Compared with the ASCI group and the ASCI+NC inhibitor group, HIF-1*α* expression in the ASCI+EZH2 inhibitor group was decreased, which showed that downregulation of EZH2 inhibits HIF-1*α* expression in ASCI rats (fold change ≈ 0.5 ~ 3; ^∗^*P* < 0.05 vs. Sham group; ^#^*P* < 0.05 vs. ASCI group; Figure 5(d)).

ELISA detected the levels of IL-6, TNF-*α*, and IL-17 in the cerebrospinal fluid of rats in each group. It was observed that EZH2 could inhibit the levels of IL-6, TNF-*α*, and IL-17 in the ASCI group (fold change ≈ 0.5 ~ 4; ^∗^*P* < 0.05 vs. Sham group; ^#^*P* < 0.05 vs. ASCI group; Figures 5(e)–5(g)). As shown in Figure 5(h), metabolomics results indicated that adding EZH2 inhibitor GSK126 inhibits the relative levels of glycolytic metabolites G6P, F6P, SUCCINATE, and MALATE in the ASCI group. Supplementary Figure 1 shows the relationship diagram where EZH2 mediates miR-146a-5p/HIF-1*α* to alleviate inflammation and glycolysis. The above results suggest that EZH2 mediates miR-146a-5p/HIF-1*α* to relieve inflammation and glycolysis in ASCI.

## 4. Discussion

ASCI has a high rate of disability and mortality. For ASCI patients with incomplete spinal cord injury, early reasonable diagnosis and treatment measures can promote nerve recovery to a certain extent and improve the prognosis of patients [24]. Studies have shown that immune inflammation and glycolysis play an important role in the progression of ASCI [25, 26]. But the specific mechanism has not been explored clearly. In the present study, we studied the relationship between inflammation, glycolysis, HIF-1*α*, and EZH2 in microglia treated with LPS and in the ASCI animal model. The results showed that EZH2 mediates miR-146a-5p/HIF-1*α* to alleviate inflammation and glycolysis.

In the early stage of SCI, the cytokines mediating inflammation at the site of injury could secrete related inflammatory factors. These inflammatory factors then accumulate and invade the SCI injury site, eventually leading to a secondary inflammation that aggravates the occurrence of injury, including TNF-*α* and IL-6 [27]. TNF-*α* is an important inflammatory mediator and pathogenic factor, which can promote cells and tissues in the CNS to produce other inflammatory mediators together with leukocytes and participate in the inflammatory response [28]. In the early stage of SCI, the increase of IL-6 can cause inflammation and edema in the injured area, aggravate the infiltration of inflammatory cells, and inhibit the recovery of nerve function [29, 30]. Studies have shown that IL-17 can stimulate astrocytes to release chemokines, causing the infiltration of peripheral neutrophils into the CNS and aggravating the inflammatory response [31]. We found that knockdown of EZH2 could effectively inhibit the synthesis and release of TNF-*α*, IL-6, and IL-17.

The main function of EZH2 is to methylate H3K27me3 by transferring the methyl from the cofactor S-adenosine L-methionine (SAM), but H3K27me3 is associated with gene transcription inhibition, so EZH2 cannot directly act on HIF-1*α* through H3K27me3. We hypothesized that EZH2 may indirectly regulate HIF-1*α* expression by mediating the methylation of miRNA upstream of HIF-1*α*. It was found that after interference with EZH2 and overexpression of HIF-1*α*, the release of inflammatory factors TNF-*α*, IL-6, and IL-17 is reduced, thus alleviating the damage of inflammatory response caused by ASCI, which indicates that EZH2 can regulate the inflammation through HIF-1*α*. Studies have suggested that miR-146a-5p is downregulated after the occurrence of ASCI [16], and studies have also verified that miR-146a-5p has a targeting relationship with HIF-1*α* [17]. We confirmed that EZH2 could indirectly regulate HIF-1*α* expression by mediating miR-146a-5p after addition of EZH2 and miR-146a-5p inhibitors.

Increased glycolysis has also been found in a variety of neurological diseases, such as cerebral ischemia, neurodegenerative diseases, traumatic brain injury, and SCI [26, 32–34]. Previous studies have shown that increased glycolysis may provide a potential source of energy for the CNS, which can rescue nerve cells [35]. HIF-1 is a key factor in glycolysis, which stimulates the expression of glycolysis transporters and enzymes supporting high glycolysis rates [34]. The effective self-activation of HIF-1 is achieved via its interaction with glycolytic pathways [8]. Under hypoxia, HIF-1, which is stable in cancer cells, promotes glycolytic activity through upregulating GLUT1 and PKM2 expression [36]. The upregulation of PKM2 in turn promotes the transactivation of HIF-1, and the glycolysis product lactic acid further stabilizes HIF-1, therefore forming a self-enhancing feedback loop for HIF-1 activity [34]. PKM2 has been reported to be a key determinant of LPS activating macrophages and promoting inflammatory responses [37]. Inhibition of HIF-1 promotes apoptosis through HK2-dependent mechanisms and reduces systemic vascular remodeling disease [38]. Previous studies have found that EZH2 could promote tumorigenesis and malignant progression via activating glycolysis of EAF2-HIF-1*α* signaling transduction [39]. We found that after interference with EZH2 and overexpression of HIF-1*α*, glycolysis-related gene PKM2, GLUT1, and HK2 expressions decreased, indicating that EZH2 could regulate glycolysis through HIF-1*α*. In the ASCI animal model, we also found that EZH2 regulates glycolysis by mediating miR-146a-5p/HIF-1*α*. In summary, we found that EZH2 mediates miR-146a-5p/HIF-1*α* to alleviate the inflammation and glycolysis after ASCI. However, this study is only a preliminary study. Although our research can illustrate the purpose of the study, our Western blot experimental results also have some imperfections. For example, the double banding of H3 and IL-6 in Figure 4 may be caused by the high concentration of the primary or secondary antibody, the nonspecific reaction of the polyclonal antibody itself, the weak specificity of the antibody, or the long compression time of the membrane. In addition, further experiments will be conducted in the future to study the role of epigenetic regulation in ASCI.

## 5. Conclusion

Our research results demonstrated that EZH2 mediates miR-146a-5p/HIF-1*α* to alleviate the inflammation and glycolysis after ASCI. Our study provides reference and basis for the clinical treatment and prognosis of ASCI in the future and provides a new target for the treatment of ASCI.

## Figures and Tables

**Table 1:** The primer sequences used in this study.

Primer ID	5'-3'
EZH2-F	TCTCACCAGCTGCAAAGTGT
EZH2-R	AGAGGAGTTGTGTTTTCCCACT
HIF-1*α*-F	ACGATTGTGAAGTTAATGCTCCC
HIF-1*α*-R	AACCAACAGAAACGAAACCCC
IL-6-F	TCACTATGAGGTCTACTCGG
IL-6-R	CATATTGCCAGTTCTTCGTA
TNF-*α*-F	CCCCTCTATTTATAATTGCACCT
TNF-*α*-R	CTGGTAGTTTAGCTCCGTTT
IL-17-F	CGTTTCCTCTATTGTCCGCCAT
IL-17-R	TGGAAGGCAGACAATTCTAACCC
PKM2-F	GTGCCGCCTGGACATTGACTC
PKM2-R	ATTCAGCCGAGCCACATTCATCC
GLUT1-F	GCTGTGGCTGGCTTCTCTAACTG
GLUT1-R	AGCAGCACCGTGAAGATGATGAAG
HK2-F	CCTGGTTTCAAAGCGGTCG
HK2-R	AGTCGGGGTCGAGTAGAGAAA
miR-146a-5p-R	GCTGTCAACGATACGCTACGTAAC
miR-146a-5p-F	AAGTTCAGTTCTTTGTCGACGTA
5S-F	GCCTACAGCCATACCACCCGGAA
5S-R	CCTACAGCACCCGGTATCCCA
*β*-Actin-F	ACATCCGTAAAGACCTCTATGCC
*β*-Actin-R	TACTCCTGCTTGCTGATCCAC

## Data Availability

All data and figures used to support the findings of this study are included within the article.

## Abbreviations

ASCI: Acute spinal cord injury
LPS: Lipopolysaccharide
qRT-PCR: Quantitative real-time PCR
CNS: Central nervous system
EZH2: Enhancer of zeste homology 2
PRC2: Polycomb repressive complex 2
H3K27me3: Histone H3 lysine 27 trimethylation
HIF-1: Hypoxia inducible factor 1
SCI: Spinal cord injury
miRNA: MicroRNA
BBB: Basso-Beattie-Bresnahan
ChIP: Chromatin immunoprecipitation
WT: Wild type
MUT: Mutant
GC-TOFMS: Gas chromatography time-of-flight mass spectrometry
G6P: Glucose-6-phosphate
F6P: Fructose-6-phosphate
SD: Standard deviation
SAM: S-Adenosine L-methionine.

## Supplementary Materials

Supplementary Materials Supplementary Figure 1: the relationship diagram about EZH2 mediates miR-146a-5p/HIF-1*α* to alleviate inflammation and glycolysis.
