# Supplementary figures and images for: EZH2 Mediates miR-146a-5p/HIF-1α to Alleviate Inflammation and Glycolysis after Acute Spinal Cord Injury
Source: Mediators Inflamm. 2021 May 19;2021:5591582. doi: 10.1155/2021/5591582 (PMC8159642; doi:10.1155/2021/5591582)

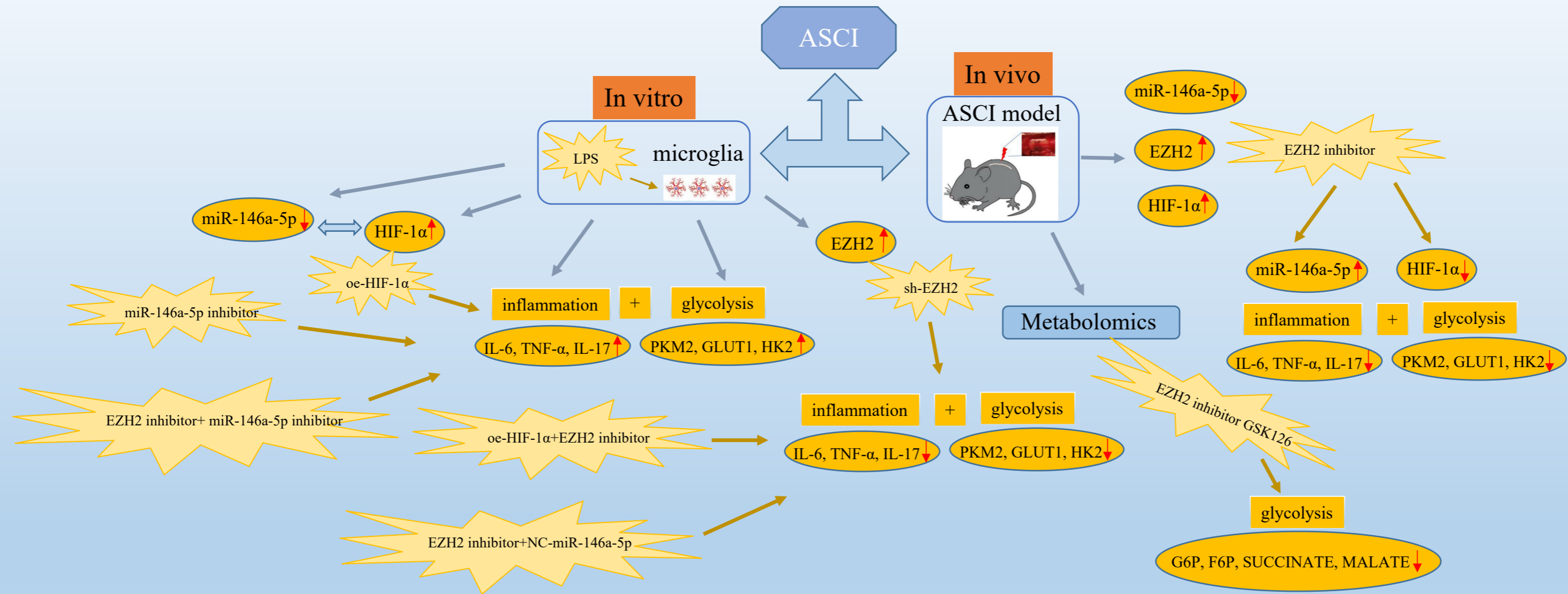

Supplement: Supplementary Materials — Supplementary Figure 1: the relationship diagram about EZH2 mediates miR-146a-5p/HIF-1α to alleviate inflammation and glycolysis. [file 5591582.f1.pdf]
